# Potent ABA‐independent activation of engineered PYL3

**DOI:** 10.1002/2211-5463.13151

**Published:** 2021-04-07

**Authors:** Yutao Wang, Chong Feng, Xiangtao Wu, Weihong Lu, Xiaoli Zhang, Xingliang Zhang

**Affiliations:** ^1^ Guangdong Provincial Key Laboratory of Biotechnology for Plant Development and Guangzhou Key Laboratory of Subtropical Biodiversity and Biomonitoring School of Life Sciences South China Normal University Guangzhou China; ^2^ Department of Biological Food and Environment Hefei University China; ^3^ Department of Pediatrics The First Affiliated Hospital of Xinxiang Medical College Weihui China; ^4^ Institute of Pediatrics Department of Hematology and Oncology Shenzhen Children’s Hospital China; ^5^ Department of Pediatrics The Affiliated Hospital of Guangdong Medical University Zhanjiang China; ^6^ State Key Laboratory of Agrobiotechnology College of Biological Sciences China Agricultural University Beijing China

**Keywords:** ABA independent, ABA irresponsive, constitutive inhibition, HAB1, PYL3

## Abstract

Abscisic acid (ABA) plays a vital role in many developmental processes and the response to adaptive stress in plants. Under drought stress, plants enhance levels of ABA and activate ABA receptors, but under harsh environmental stress, plants usually cannot efficiently synthesize and release sufficient quantities of ABA. The response of plants to harsh environmental stress may be improved through ABA‐independent activation of ABA receptors. The molecular basis of ABA‐independent inhibition of group A protein phosphatases type 2C (PP2Cs) by pyrabactin resistance/Pyr1‐like (PYR1/PYLs) is not yet clear. Here, we used our previously reported structures of PYL3 to first obtain the monomeric PYL3 mutant and then to introduce bulky hydrophobic residue substitutions to promote the closure of the Gate/L6/CL2 loop, thereby mimicking the conformation of ABA occupancy. Through structure‐guided mutagenesis and biochemical characterization, we investigated the mechanism of ABA‐independent activation of PYL3. Two types of PYL3 mutants were obtained: (a) PYL3 V108K V107L V192F can bind to ABA and effectively inhibit HAB1 without ABA; (b) PYL3 V108K V107F V192F, PYL3 V108K V107L V192F L111F and PYL3 V108K V107F V192F L111F cannot recognize ABA but can greatly inhibit HAB1 without ABA. Intriguingly, the ability of PYL3 mutants to bind to ABA was severely compromised if any two of three variable residues (V107, V192 and L111) were mutated into a bulky hydrophobic residue. The introduction of PYL3 mutants into transgenic plants will help elucidate the functionality of PYL3 *in vivo* and may facilitate the future production of transgenic crops with high yield and tolerance of abiotic stresses.

## Graphical abstract

PYL3 binds to abscisic acid (ABA) to inhibit the phosphatase activity of PP2Cs effectively. The molecular basis of ABA‐independent inhibition of PP2Cs by PYL3 is not yet clear. Here, we first obtain the monomeric PYL3 mutant and then introduce bulky hydrophobic residue substitutions to promote closure of the Gate/L6/CL2 loop. Four PYL3 mutants can greatly inhibit PP2Cs without ABA.

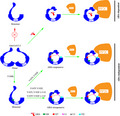

**Abbreviations**
ABA: abscisic acid
CA: constitutively active
EGS: ethylene glycol‐bis (succinic acid *N*‐hydroxysuccinimide ester)
ITC: isothermal titration calorimetry
PP2Cs: group A protein phosphatases type 2C
PYR1/PYL/RCAR: pyrabactin resistance/Pyr1‐like/regulatory components of ABA receptor
SEC: size exclusion chromatography
WT: wild‐type

Abscisic acid (ABA) was first discovered in the 1960s, and its chemical structure was determined by chemical analysis. Shortly after the discovery of ABA, many of its critical physiological functions were identified. ABA is an essential phytohormone synthesized in plants that regulates many aspects of plant growth and development, such as root growth, stomatal aperture, seed maturation and dormancy [1, 2]. Moreover, ABA is also involved in regulating plant responses to abiotic stresses, including drought, cold and salinity [3, 4]. Besides, ABA can regulate fruit development [5, 6] and play a critical role in stimulating sugar accumulation and improving the yield and quality of fruit [7, 8, 9]. The mutant plants with severe ABA deficiency or ABA desensitization showed malnutrition under ideal water conditions, and seed formation was severely stunted [10, 11, 12, 13].

ABA accumulates under stress conditions and plays an essential role in the stress response and tolerance of plants [14]. When suffering from drought stress, plants enhance ABA level and activate ABA receptors by forming an ABA‐receptor complex [15]. In ABA signal transduction, recognition of ABA by its receptor is very important. If ABA receptors are dysfunctional, cells cannot recognize ABA; even in the presence of high ABA concentrations, the corresponding physiological response will not occur. However, an increase in the number of ABA receptors can enhance the cells’ sensitivity to ABA, enabling the plants to quickly acquire resistance to environmental stresses under a low ABA concentration. Nevertheless, in the face of various harsh environmental stresses, plants usually cannot efficiently synthesize and release sufficient quantities of ABA, making plants unable to respond to these stresses that are lowering resistance. Therefore, the potently constitutively active (CA) PYLs transgene probably promotes plant tolerance to extreme environmental stress. A 14‐member family of pyrabactin resistance/Pyr1‐like/regulatory components of ABA receptor (PYR1/PYL/RCAR) in *Arabidopsis thaliana* was identified to be ABA receptors (hereafter referred to as PYR/PYLs) [12, 16], except PYL10 [17, 18] and PYL13 [19, 20], which must bind to ABA to inhibit the phosphatase activity of PP2Cs effectively [21]. PYR1 was subjected to site‐saturation mutagenesis to increase basal PYR1–PP2C interactions, which generated three fully CA PYR1 mutants (PYR1^CA3^, PYR1^CA4^ and PYR1^CA4B^) and homologous fully CA PYL2 and PYL9 mutants [22], as well as the related invention [23]. Therefore, it is imperative to manufacture other PYR/PYLs proteins that can robustly inhibit the activity of downstream protein PP2Cs in the absence of ABA.

PYR1 [24, 25], PYL1 [26], PYL2 [27], PYL3 [28, 29], PYL5 [30], PYL9 [30], PYL10 [17, 18] and PYL13 [19] were characterized by structural and biochemical studies, which convincingly documented an ABA‐dependent signaling mechanism. On ABA binding, several loops surrounding the ligand‐binding pocket of PYLs, particularly the Gate/L6/CL2 loop, underwent noticeable conformational changes [27, 28, 29]. The newly created surface was precisely designed for interaction with PP2Cs, including ABI1, ABI2 and HAB1. Formation of the ternary complex PYLs–ABA–PP2C blocked natural substrates to bind PP2Cs, which released PP2C‐mediated inhibition of the downstream substrates SnRK2 kinases. Therefore, from the ABA‐dependent signaling mechanism, two prerequisites for the ABA‐independent inhibition of PP2Cs by PYLs must be simultaneously satisfied. First, PYLs must be in a monomer state because the interface of heterodimeric PYLs–PP2Cs overlaps seriously with that of dimeric PYLs. Second, the Gate/L6/CL2 loop of the ligand‐binding pocket must be in a closed state, which is essential to create a new interface for PP2Cs binding. If both of these conditions are satisfied for PYLs without ABA, the constitutive inhibition of HAB1 can occur.

Here, based on our previously reported crystal structures of apo‐PYL3, PYL3–ABA and PYL3–ABA–HAB1 [28], the engineered PYL3 monomeric mutants with the nearly intact ability of binding to ABA were obtained by mutagenesis. Then, the bulky hydrophobic residues substitutions coordinating the Gate/L6/CL2 continued to be introduced to promote the closure of the Gate/L6/CL2, which could potently inhibit the phosphatase activity of HAB1 in the absence of ABA. Holistically, our results will provide an essential basis for understanding the ABA‐independent inhibition of HAB1 by engineered PYL3. The potently CA PYL3 mutant transgene could not only activate PYL3 selectively and dissect phenotypic consequences but also promote plant tolerance to extreme abiotic stress.

## Materials and methods

### Protein preparation

The wild‐type *PYL3* (*PYL3* WT) were subcloned from the *Arabidopsis thaliana* cDNA library using a standard PCR‐based protocol. The *PYL3* WT was inserted into the pET‐28a vector (Novagen), in which the TEV recognition site replaced the thrombin recognition site (hereafter termed as pET‐28a‐tev). All PYL3 mutants were generated by circular site‐directed mutagenesis technology [31, 32]. In short, according to the gene sequence of *PYL3*, the target mutation site is in the middle of the complementary primers region, and there is 20–30 bp complementation in the primer sequence pair with 8–10 bp in each 5′ end (Table S1). *PYL3* circular plasmid was used as a template for PCR amplification. The template plasmid was then digested by restriction endonuclease *Dpn*I and transformed into DH5α‐competent cells for gap repair. Finally, the site‐directed mutant plasmid of the target protein was obtained. The truncated ABI1 (104–434) and ABI2 (101–423) were subcloned from the *Arabidopsis thaliana* cDNA library and then inserted into pETT‐28a‐tev. All primers are listed in Table S1. All inserted sequences were verified by DNA sequencing.

Proteins were purified according to the protocol described previously [28, 30]. In brief, all of the proteins were overexpressed in *Escherichia coli* strain BL21(Rosseta), which were induced with IPTG at 18 °C for 24 h. Cells were harvested by centrifugation at 4000 ***g*** for 30 min and then resuspended in buffer (20 mm Tris–HCl [pH 8.0], 150 mm NaCl) and lysed by sonication. The ABI1 (104–434), ABI2 (101–423), PYL3 and its mutated proteins were purified with Ni‐charged resin (Bio‐Rad, Hercules, CA, USA), followed by size exclusion chromatography (SEC; Superdex‐200; GE Healthcare, Pittsburgh, PA, USA). HAB1 (169–511) from the *Arabidopsis thaliana* cDNA library was inserted into the pGEX‐4T‐2 vector (GE Healthcare) in which the tobacco etch virus (TEV) protease recognition site replaced the thrombin recognition site. The Glutathione S‐transferase (GST)‐tagged protein was applied to glutathione sepharose resin (GE Healthcare). Then the 6 × His‐tagged TEV protease was added into the glutathione sepharose resin‐loading column to excise GST‐tag.

### Phosphatase activity assay

The phosphatase activity was measured by the Serine‐Threonine Phosphatase Assay System Kit (V2460; Promega, Madison, WI, USA), whose protocol was described previously with some modifications [28, 30]. In brief, HAB1 was adjusted to their indicated concentrations to warrant the assay’s readout within the rational range. A total of 1 μm PYL3 WT or mutant proteins and ABA with 5 molar equivalents of protein concentration, if required, were gently mixed with HAB1 in 45 μL of the final reaction volume supplemented with reaction buffer (20 mm Hepes [pH 7.5] and 150 mm NaCl) and then incubated at 30 °C for 30 min. A total of 5μL phosphopeptide substrate (1 mm) supplied with the kit was added into the reaction system in a water bath of 30 °C for 25 min. The reaction was terminated by the addition of 50 μL molybdate dye/additive mixture, and the absorbance at 630 nm (*A*_630 nm_) was measured 30 min later. The readout of reaction solution without PP2Cs was set as the baseline, which was subtracted from all the assay data. Data were analyzed with GraphPad Prism 5.0 and expressed as mean ± standard deviation from at least three independent experiments.

### SEC

The 6× His‐tagged PYL3 and their mutant proteins were applied to Superdex‐200 (GE Healthcare) in a buffer containing 20 mm Hepes or PBS (pH 7.5) and 150 mm NaCl. The fractions were visualized by SDS/PAGE, followed by Coomassie blue staining to estimate their purity.

### Isothermal titration calorimetry assays

Isothermal titration calorimetry (ITC) is a technique used to study the interaction of various biomolecules quantitatively. It can directly measure the heat released or absorbed during the binding process of biomolecules. All proteins were subjected to SEC with buffer A (20 mm Tris–HCl [pH 8.0] and 150 mm NaCl). A total of 100 mm ABA was dissolved in buffer A and adjusted to pH 8.0 with NaOH. A total of 3 mm ABA (Sigma, St. Louis, MO, USA) was obtained by diluting 100 mm stock solution with buffer A. A total of 3 mm ABA in the syringe was titrated against 0.22 mm WT or mutant PYL protein in the cell. Buffer A without ABA was used as a negative control. The temperature of the reaction system was set at 18 °C. The experiments were performed with Nano‐2G‐ITC (TA Corporation, New Castle, DE, USA), and the thermodynamic parameter was determined using the software included with the specific instrument, as given in our previous reports [33].

### Cross‐linking gel assay

Cross‐linking gel assay by ethylene glycol‐bis (succinic acid *N*‐hydroxysuccinimide ester) (EGS) (E‐3257; Sigma) was used to detect the oligomeric state of the proteins, as described previously [28, 30]. After the SEC’s purification, 20 μg 6× His‐tagged PYL3 WT or mutant proteins in PBS (pH 7.5, 150 mm NaCl) was added to 100 μL of reaction volume. The reaction was maintained for 10 min at room temperature; then 5 μL of 1 M Tris–glycine (pH 7.5) was added to terminate the reaction. After boiling for 5 min in the loading buffer, a small number of samples was subjected to 10% SDS/PAGE and stained with Coomassie brilliant blue.

### Yeast two‐hybrid experiment

The interactions of PYL3 or its mutants with HAB1 were detected by Matchmaker Two‐Hybrid Systems 2 (Clontech, San Francisco, CA, USA). Using PYL3 and its mutants as templates, we obtained the PCR products (including PYL3 WT, PYL3 V108K V107L V192F, PYL3 V108K V107F V192F, PYL3 V108K V107L V192F L111F and PYL3 V108K V107F V192F L111F) by forward primer 5′‐CT*ctcgag*AGATGAATCTTGCTCCAATCC‐3′ and reverse primer 5′‐CACG*ctcgag*TCAGGTCGGAGAAGCCGT‐3′. Each of the PCR products was digested by *Nco*I/*Xho*I enzymes and then was ligated to pGBKT7 plasmid to produce bait (BD‐PYL3 WT, BD‐PYL3 V108K V107L V192F, BD‐PYL3 V108K V107F V192F, BD‐PYL3 V108K V107L V192F L111F and BD‐PYL3 V108K V107F V192F L111F). HAB1 (169–511) was subcloned into a pGADT7 vector (AD‐HAB1) as prey by forward primer 5′‐CT*ctcgag*AGATGAAAATAGTAATCATCTGGTG‐3′ and reverse primer 5′‐CA*ctcgag*TCAGGTTCTGGTCTTG‐3′. Then, BD‐PYL3 or mutants and AD‐HAB1 were transformed into *Saccharomyces cerevisiae* AH109 for coexpression. AD‐HAB1 and pGBKT7 empty vector (or BD‐PYL3 and pGADT7 empty vector) were used to identify their self‐activations. The transformed yeast cells grew on SD‐TL (Trp^‐^, Leu^‐^) or SD‐TLHA (Trp^‐^, Leu^‐^, His^‐^, Ade^‐^). The colonies growing on the SD‐TLHA plate were selected and transferred to an SD‐TLHA liquid medium. After cultivation for approximately 2 days, the concentration of each sample was *A*_600_ = 0.5. The bacterial solution was diluted five times with sterilized water, and a 10 μL diluted bacterial solution was dotted on an SD‐TLHA agarose plate (100 μL of 5 μm ABA, if needed, was added to the bacterial dot on the SD‐TLHA twice for 1 day). Bacterial growth on the plate can be observed after 2 or 3 days at 30 °C.

## Results

### The mutated PYL3 monomer retained the ability to inhibit HAB1

**Fig. 1:**
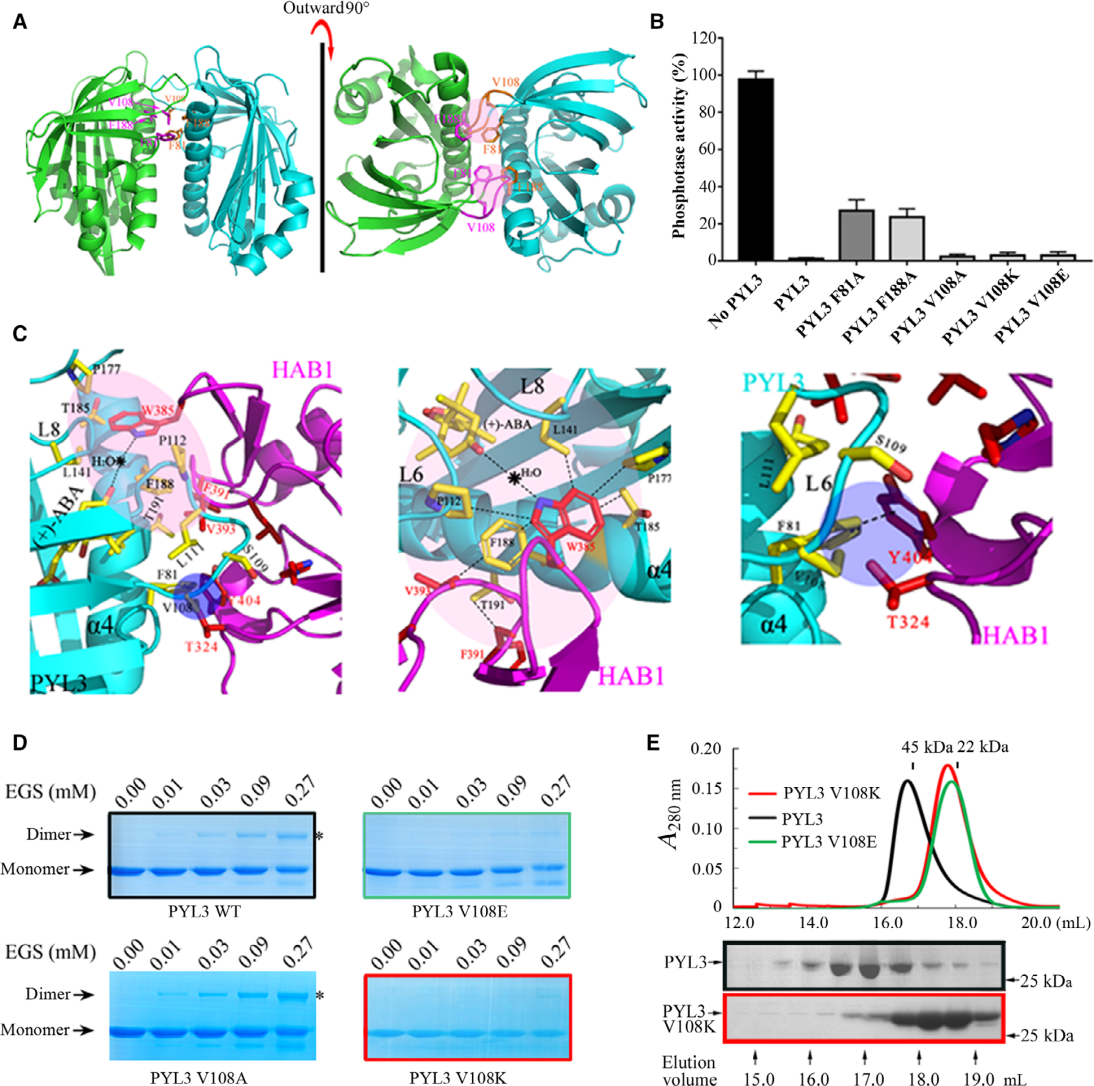
Monomeric PYL3 mutant proteins retain the ability to inhibit HAB1 activity and bind to ABA. (A) The interactions between two apo‐PYL3 protomers were mainly mediated by F81, V108 and F188. (B) The abilities of PYL3 and its mutant proteins (1.0 μm) to inhibit HAB1 (0.5 μm) activity in the presence of ABA (5 μm) were detected by the Serine‐Threonine Phosphatase Assay System Kit. Data were expressed as mean ± standard error of the mean. (C) The heterodimer interface interactions in the PYL3–ABA–HAB1 structure were analyzed. F81 and F188 were the hydrophobic interaction centers, whereas V108 contributed little to the hydrophobic interaction. (D) EGS was used to detect the monomer or dimer state of a protein. (E) SEC assays confirmed that the point mutation of V108K or V108E could destroy the interface interactions of dimeric PYL3 and produce the monomeric forms.

Based on our previously reported crystal structure of PYL3–ABA–HAB1 [28], the dimeric PYL3 must bind to ABA and then inhibit the downstream substrate HAB1 (Fig. S1). Disrupting the dimer formation was the primary mission to obtain the PYL3 mutants that do not depend on ABA to efficiently inhibit PP2Cs. The interaction network of the dimeric interface in the apo‐PYL3 crystal structure was analyzed. The key hydrophobic interaction centers were mediated by F81, V108 and F188 (Fig. 1A). First, F81, V108 and F188 were mutated to Ala (Table S1), which destroyed the hydrophobic interaction between two protomers. The ability of the mutant protein to inhibit HAB1 in the presence of ABA was detected. PYL3 V108A, PYL3 V108K and PYL3 V108E retained almost the same level of inhibition of HAB1 as PYL3 WT, but PYL3 F81A and PYL3 F188A could only inhibit about 70% of the activity of HAB1 (Fig. 1B). According to the crystal structure of PYL3–ABA–HAB1, the interface interactions between PYL3 and HAB1 heterodimer were analyzed. F188 and F81 were the hydrophobic interaction centers between PYL3 and HAB1. F188 formed a hydrophobic force net with V393, W385 and F391 in HAB1, F81 forms hydrophobic force with Y404 in HAB1, whereas V108 contributes little to the hydrophobic interaction dominated by F81. The small contributions to the hydrophobic center of PYL3 and the HAB1 heterodimer interface were mainly attributed to the V108 located flexible loop (Fig. 1C). A cross‐linking gel assay was then used to detect whether the point mutations of V108A, V108K and V108E successfully changed the dimeric PYL3 protein into the monomeric state. With the increase of EGS concentration, the dimer of PYL3 WT and PYL3 V108A increased significantly, while the two mutant proteins, PYL3 V108K and PYL3 V108E, mostly remained in the monomer state (Fig. 1D). The SEC’s results further confirmed that both PYL3 V108K and PYL3 V108E mutant proteins in solution existed in monomeric form (Fig. 1E).

### The key mutations around the PYL3 pocket’s entrance promoted the ABA‐independent inhibition of HAB1

**Fig. 2:**
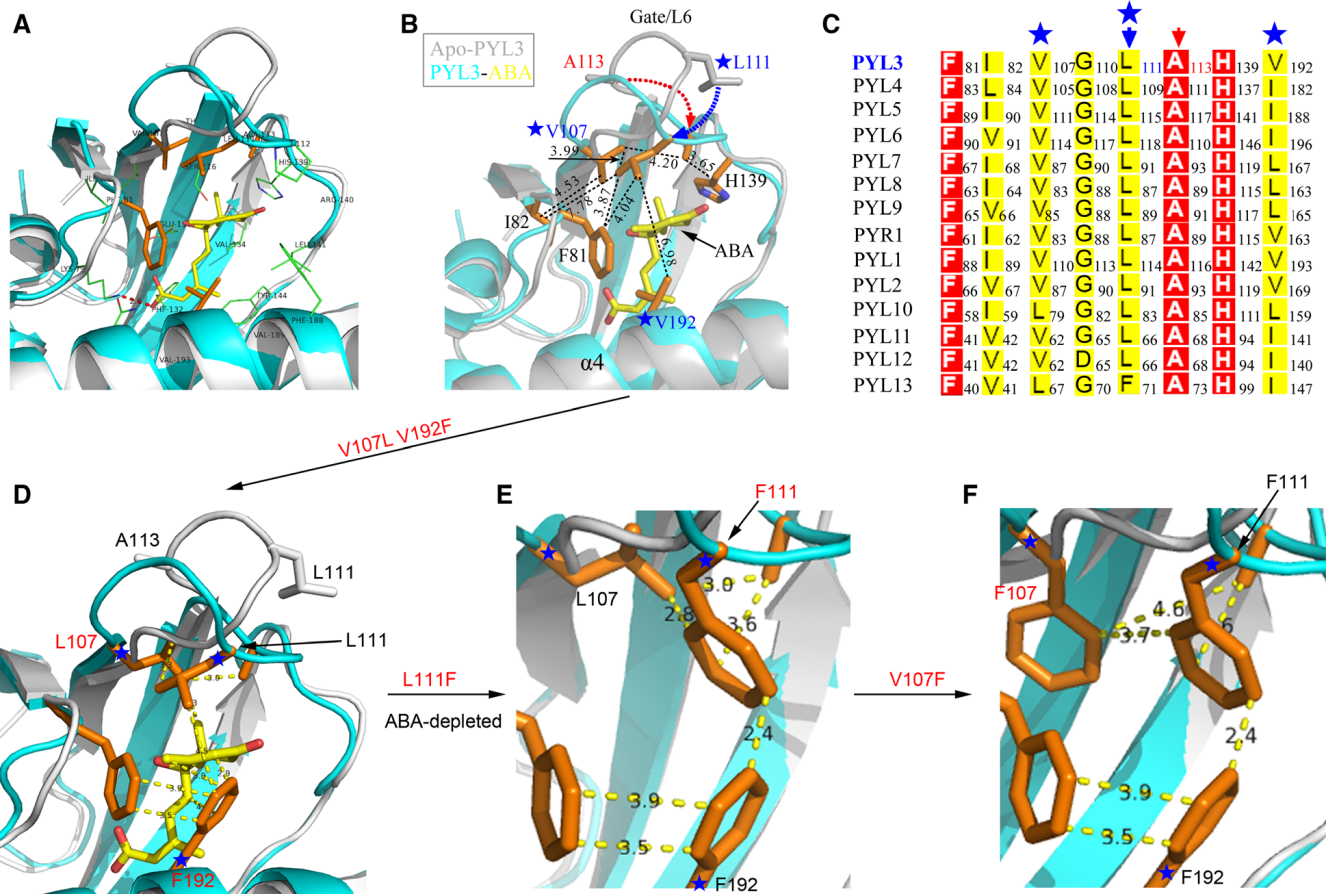
The site mutations around the PYL3 pocket’s entrance. (A) Superposition of apo‐PYL3 (gray cartoon, PDB: 3KLX) and PYL3‐ABA (cyan cartoon, PDB: 4DSC) structures. ABA (yellow stick) binds to the PYL3 pocket, which has hydrophobic interaction with many amino acid side chains inside and at the entrance of the PYL3 pocket. The Gate/L6/CL2 is closed, which is the most conformational change. In PYL3‐ABA, all side chains within 5 Å from ABA were displayed by cyan line or orange stick. The red dotted line represents the salt bond. (B) ABA binding to PYL3 leads to the maximum conformational change of L111 and A113 on Gate/L6/CL2. F81, V107, H139 and V192 could provide hydrophobic interaction with L111 and A113. (C) The amino acids in 14 PYLs family members involved in hydrophobic interactions, as shown in (B), were performed with sequence alignment (Fig. S2). (D) V107 and V192 were mutated into Leu or Phe with a larger hydrophobic group. (E) The L111F mutation was further introduced based on PYL3 V107L V192F. (F) The V107F mutation was additionally introduced based on PYL3 V107L V192F L111F. The number on the dotted line in (B) and (D)–(F) represents the distance (Å).

From the superposition comparison of apo‐PYL3, PYL3–ABA and PYL3–ABA–HAB1 structures, ABA binding has the most significant effect on the conformation of Gate/L6/CL2 at the entrance of the ABA‐binding pocket because ABA provides the critical hydrophobic force network for the closed conformation of Gate/L6/CL2 (Fig. 2A). Compared with apo‐PYL3, there are two amino acids on the Gate/L6/CL2 of PYL3‐ABA, L111 and A113, pointing to the inside of the pocket (Fig. 2B,C). Therefore, to obtain the closed Gate/L6/CL2 without ABA, the interaction intensity between L111 or A113 and the coordinating partners needs to be enhanced by mutagenesis. As shown in Fig. 2B, there were hydrophobic interactions between side chains of L111 and F81, between side chains of L111 and V107, and between side chains of L111 and V192; there is hydrophobic interaction among side chains of A113, V107 and H139. These amino acids were mutated into more hydrophobic residues, which may promote the Gate/L6/CL2 to be closed in the absence of ABA. Considering that the side chains of F81 and H139 are bulky and strongly hydrophobic, V107 and V192 are considered to be mutated into more hydrophobic residues, such as L and F (Fig. 2D–F). Then, the inhibitory effects of these PYL3 mutants on the activity of HAB1 without ABA were detected.

**Fig. 3:**
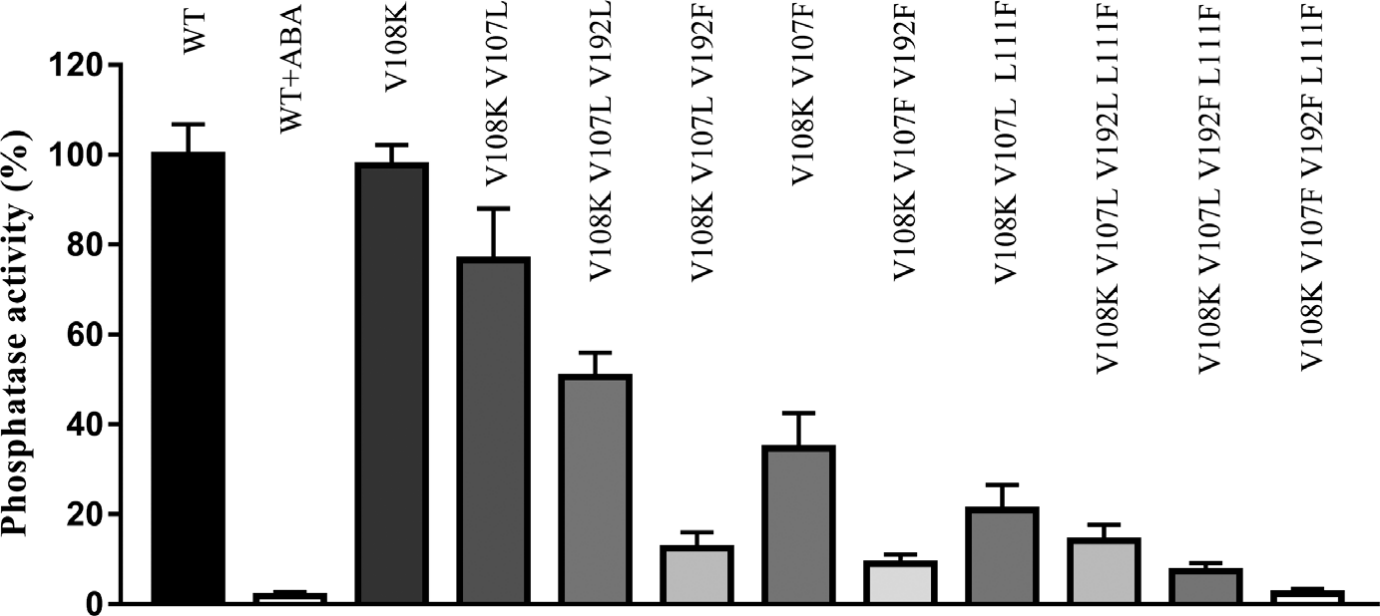
The key mutations promoted the ABA‐independent inhibition of HAB1. The abilities of PYL3 and its mutant proteins to inhibit HAB1 activity in the absence of ABA were detected by the Serine‐Threonine Phosphatase Assay System Kit. The negative control was PYL3 WT protein without ABA, and the positive control was PYL3 WT in the presence of ABA. A total of 0.5 μm HAB1 and 1 µM PYL3 mutant proteins were mixed in equal volume. Data were expressed as mean ± standard error of the mean.

PYL3 could almost completely inhibit the activity of HAB1 in the presence of ABA, but not in the absence of ABA. The monomeric PYL3 V108K was similar to PYL3 and did not possess the ability to inhibit HAB1 independent of ABA. PYL3 V108K V107L without ABA could inhibit about 25% activity of HAB1. PYL3 V108K V107L V192L without ABA could inhibit about 50% activity of HAB1. PYL3 V108K V107L V192F without ABA could inhibit about 87.5% activity of HAB1. PYL3 V108K V107F without ABA could inhibit about 65% activity of HAB1. PYL3 V108K V107F V192F without ABA could inhibit about 90% activity of HAB1. PYL3 V108K V107L L111F without ABA could inhibit about 80% activity of HAB1. PYL3 V108K V107L V192L L111F could inhibit about 85% activity of HAB1 without ABA. PYL3 V108K V107L V192F L111F could inhibit about 92.5% activity of HAB1 without ABA. PYL3 V108K V107F V192F L111F could inhibit about 98% activity of HAB1 without ABA. The earlier results are shown in Fig. 3. Importantly, these four mutated PYL3 showed strong inhibition on other clade A PP2Cs (e.g. ABI1 and ABI2) (Fig. S4). To determine whether the PYL3 mutations work in the context of other PYLs, we introduced the homologous substitutions into PYL9, generating PYL9 V85L L165F (corresponding to PYL3 V108K V107L V192F) and PYL9 V85F L89F L195F (corresponding to PYL3 V108K V107F V192F L111F). These two mutated PYL9 proteins highly inhibit HAB1 in the absence of ABA (Fig. S5). In summary, four mutant proteins, namely, PYL3 V108K V107L V192F (87.5%), PYL3 V108K V107F V192F (90%), PYL3 V108K V107L V192F L111F (92.5%) and PYL3 V108K V107F V192F L111F (98%), could strongly inhibit the activity of HAB1.

**Fig. 4:**
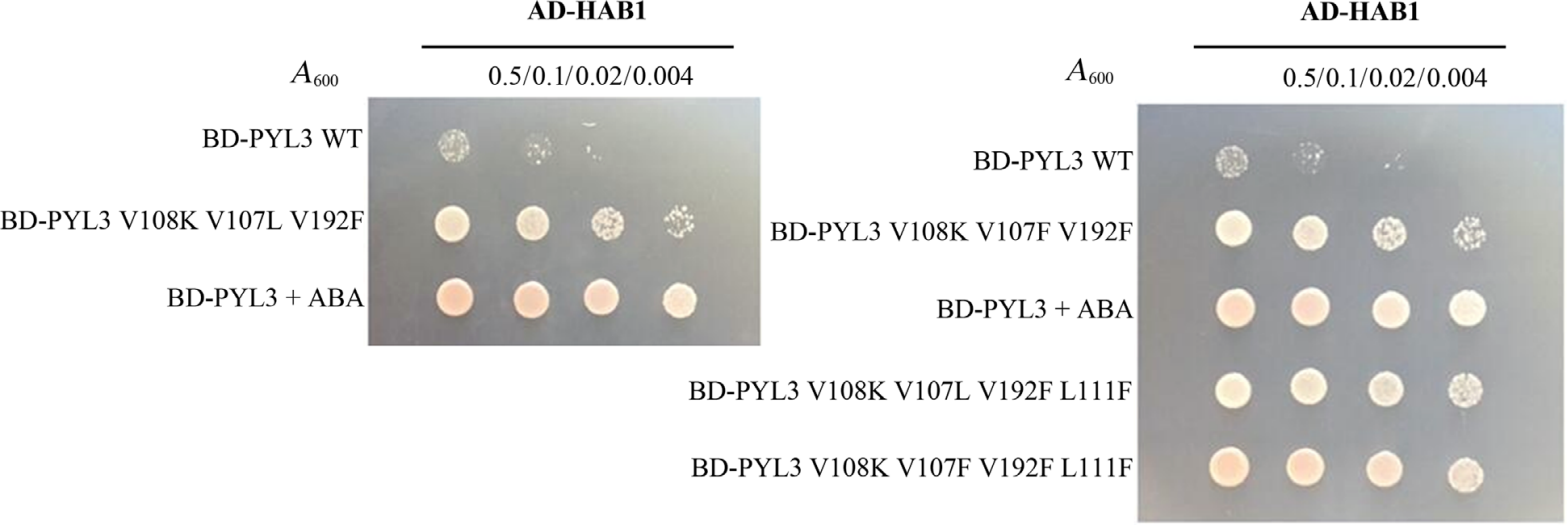
The interactions of PYL3 and its mutant proteins with HAB1 were detected by the yeast two‐hybrid system. *PYL3* and its mutant were constructed on the activation domain (AD) vector and HAB1 on the binding domain (BD) vector. Four yeast cell concentrations were selected when *A*_600 nm_ was 0.5, 0.1, 0.02 and 0.004. A total of 100 μL of 5 μm ABA solution was added to the yeast plaque center every 12 h for 2–3 days if needed.

Because the family of PYR/PYLs proteins inhibits HAB1 activity by directly binding and blocking HAB1 enzyme activity center entrance, the interaction between PYL3 mutant protein and HAB1 is positively related to the effect of PYL3 mutant protein inhibiting HAB1 activity. Therefore, the interaction between each of four potent PYL3 mutant proteins and HAB1 was further detected by the yeast two‐hybrid technique. As shown in Fig. 4, PYL3 WT can efficiently bind to HAB1 in the presence of ABA, but not in the absence of ABA. PYL3 V108K V107L V192F and PYL3 V108K V107F V192F without ABA showed good binding abilities to HAB1. PYL3 V108K V107L V192F L111F and PYL3 V108K V107F V192F L111F showed the most robust constitutive interaction with HAB1.

### The key mutations are the determinators of ABA‐irresponsive inhibition of HAB1

**Fig. 5:**
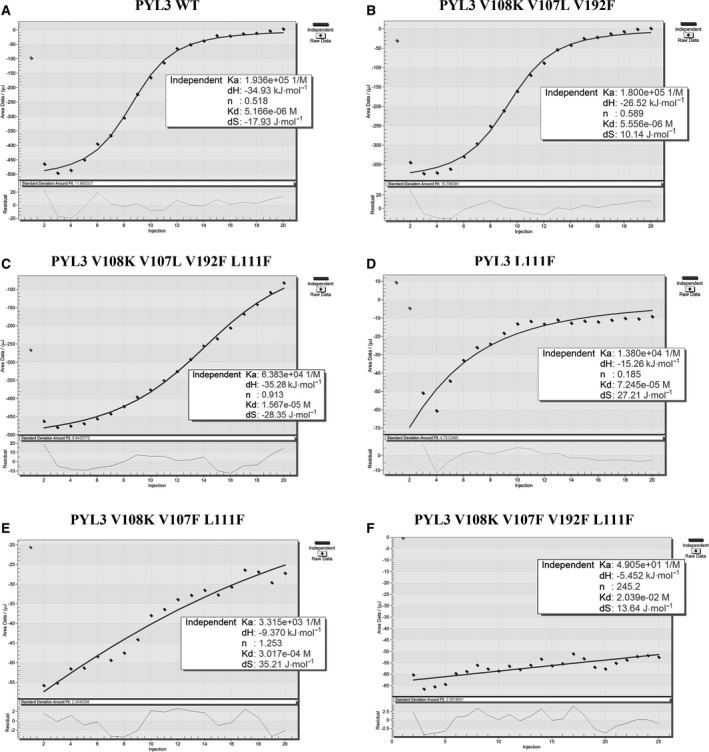
Isothermal titration calorimetric analysis of the binding of ABA to PYL3 mutants. The binding abilities of (A) PYL3 WT, (B) PYL3 V108K V107L V192F, (C) PYL3 V108K V107L V192F L111F, (D) PYL3 L111F, (E) PYL3 V108K V107F L111F and (F) PYL3 V108K V107F V192F L111F to ABA were detected by ITC.

The ITC assays were used to detect whether the four PYL3 mutants with potent constitutive inhibition on HAB1 retained the same ability of PYL3 WT to recognize ABA. As shown in Fig. 5, PYL3 WT has a strong ABA‐binding capacity (*K*_d_ = 5.166 × 10^−6^ m). PYL3 V108K V107L V192F showed almost the same ability to bind ABA as PYL3 WT (*K*_d_ = 5.566 × 10^−6^ m). However, PYL3 V108K V107L V192F L111F showed weak ABA‐binding ability (*K*_d_ = 1.567 × 10^−5^ m). It could be inferred that the mutation L111F may damage the ability of PYL3 binding to ABA, which was confirmed by the ITC experiment of L111F (*K*_d_ = 7.245 × 10^−5^ m). PYL3 V108K V107F V192F displayed weaker ABA‐binding capacity (*K*_d_ = 3.017 × 10^−4^ m) compared with PYL3 V108K V107L V192F. So, the V107F mutation could severely impair the ability of PYL3 to bind to ABA. PYL3 V108K V107F V192F L111F could not recognize ABA (*K*_d_ = 2.039 × 10^−2^ m). We conclude that the triple mutation of V107F V192F L111F can cripple the ability of PYL3 to bind to ABA.

**Fig. 6:**
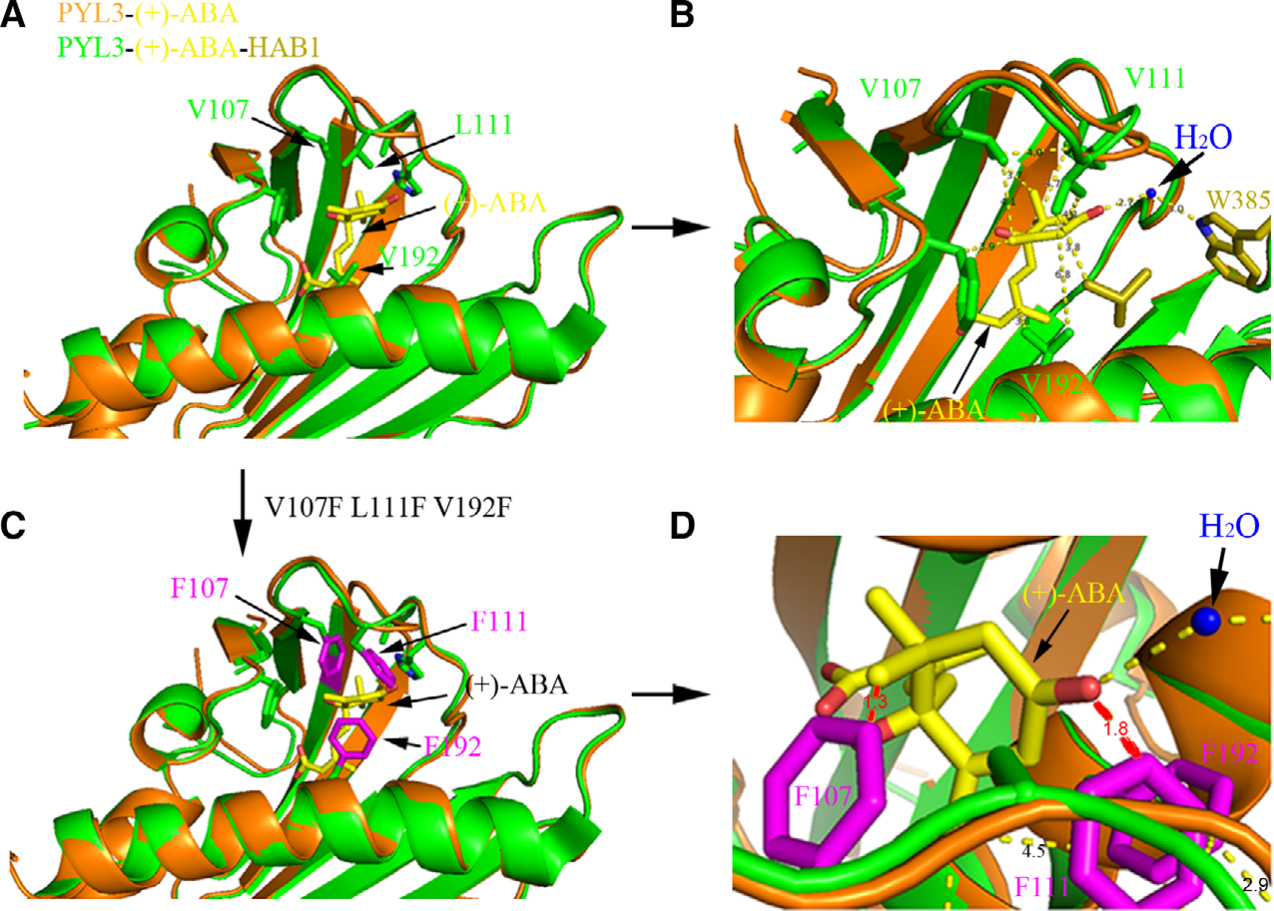
The steric hindrance between V192F, L111F or V107F mutations and ABA. (A) Structural superposition of two PYL3 protomers from PYL3–ABA (orange) and PYL3–ABA–HAB1 (PDB: 4DS8, green). (B) The residues at the pocket entrance of PYL3 WT have hydrophobic interaction with ABA. (C) V192, L111 and V107 in PYL3 were mutated into Phe. (D) There is a steric hindrance between L111F or V107F and ABA. The software PyMOL was used to measure the minimum distance from the side chain of mutated residue to ABA. (C, D) Models of PYL3 mutants.

To further elucidate the complete loss of triple‐mutation PYL3 V192F L111F V107F recognizing ABA, we mutated V192, L111 and V107 in the crystal structure of PYL3–ABA–HAB1 into Phe. We then analyzed whether ABA in the PYL3 pocket was uncomfortable with the bulky side chain of Phe for steric constraints. As shown in Fig. 6A,B, the distances between the side chains of V192, V107 or L111 and ABA were around 3–4 Å, forming the typical hydrophobic interactions. The V192F had no steric hindrance to ABA and formed a stronger hydrophobic interaction with ABA (Fig. 6C), consistent with the stronger binding of PYL3 V192F to ABA than PYL3 WT (Fig. S3). However, the minimum distance from the side chains of V107F and L111F to ABA is 1.3 and 1.8 Å, respectively (Fig. 6D). Both of these mutations have serious steric hindrance to ABA. Nevertheless, considering that L111 is located on the relatively flexible Gate/L6/CL2 loop, the steric clash with ABA by L111F becomes weaker. If V192, L111 and V107 were mutated into Phe at the same time, the side chains of these three Phe occupy the ABA‐binding pocket, resulting in the pocket unable to accommodate ABA, which is consistent with the fact that PYL3 V108K V107F V192F L111F cannot bind to ABA (Fig. 5F).

In summary, two types of PYL3 mutants were obtained in this study. PYL3 V108K V107L V192F can recognize and bind to ABA (Fig. 5) and effectively inhibit the downstream protein HAB1 enzyme activity without ABA (Figs 3 and 4). Besides, PYL3 V108K V107F V192F, PYL3 V108K V107L V192F L111F and PYL3 V108K V107F V192F L111F cannot recognize and bind to ABA (Fig. 5) and can strongly inhibit the activity of HAB1 without ABA (Figs 3 and 4).

## Discussion

Over the last several decades, there have been numerous breakthroughs in genetic and structural‐guided biochemical characterizations of ABA receptors PYR1/PYLs [34]. PYR1/PYLs proteins must bind to ABA to execute certain functions. The ABA trapped in the receptor cavity keeps the Gate/L6/CL2 in a closed state, which is a favorable conformation for PYR1/PYLs to dock into PP2Cs. ABA can normally activate several PYR1/PYLs in the WT context, and it is unclear whether different receptors bear different subfunctions. Therefore, it is of great significance to study the ABA‐independent activation of individual family members selectively by mutagenesis. Twenty‐nine mutations increasing the basal interactions between PYR1 mutants and HAB1 were screened out from all possible 741 amino acid substitutions [22]. These mutations are located in 10 residues of PYR1 (V83, I84, L87 and A89 in the Gate; M158, F159, T162, L166 and K170 in the C‐terminal helix; H60), which engage in PP2C contacts and PYR1 homodimerization. Three mutants PYR1 H60P V83F F159V (PYR1^CA3^), PYR1 H60P V83F M158I F159V (PYR1^CA4^) and PYR1 V83F M158I F159V K170W (PYR1^CA4B^) were finally confirmed to be of high constitutive inhibition on HAB1 [22]. Here, we applied a particular strategy to obtain the monomeric PYL3 mutant and then introduce bulky hydrophobic residue substitutions to promote the closure of the Gate/L6/CL2 without ABA, which could mimic the conformation of ABA occupancy. Two types of PYL3 mutants were obtained: (a) PYL3 V108K V107L V192F can bind to ABA; and (b) PYL3 V108K V107F V192F, PYL3 V108K V107L V192F L111F and PYL3 V108K V107F V192F L111F cannot recognize ABA. Both types can highly inhibit HAB1 without ABA. The ABA irresponsiveness of PYL13 was attributed to the bulky F71 on the L6/Gate/CL2 and the lack of the positive charge for the Q38 within pocket [19]. Interestingly, we found the ability of PYL3 binding to ABA was severely compromised if any two of three variable residues (V107, V192 and L111) were mutated into bulky hydrophobic residues, such as Phe. Together, we combine structure‐guided mutagenesis and biochemical characterizations to elucidate the mechanism of the ABA‐irresponsive or ABA‐responsive activation of PYL3, which may contribute to the production of the transgenic crop with high yield and tolerance of abiotic stresses by the biotechnological applications of PYR1/PYLs.

The Gate/L6/CL2 was relatively flexible and prone to be in an open state for apo‐PYL5, apo‐PYR1 and PYL1‐PYL3 [17, 35], incompatible for PP2Cs binding. Interestingly, the opened and closed gates were seized in two structures of apo‐PYL10 from two research groups [17, 18]. PYL10 added in 10‐fold molar ratios showed a high basal activity and ability to inhibit HAB1 [17]. Therefore, it is theoretically feasible to improve the Gate/L6/CL2 closure conformation of apo‐PYLs by mutagenesis. The structural and *in vitro* biochemical investigation elucidates that monomeric state and having a bulky hydrophobic residue around the ligand‐binding pocket are two molecular determinants for the high basal activity of PYL10 [17]. The key residues of PYL10 for high basal activity were incorporated into the corresponding positions of PYL2, generating the mutant PYL2 V87L I88K [17]. However, this double mutant only partially enhanced the basal activity of PYL2. PYR1 V83 is corresponding to PYL10 L79 and PYL2 V87 in sequence alignment. PYR1 V83F, but not PYR1 V83L, can significantly promote constitutive activation of ABA signaling [22]. Nevertheless, another group demonstrated that PYL10 activity was ABA dependent [36]. From the different efficiency of constitutive inhibition of HAB1 by the monomeric PYL3 mutants, we discovered that the three variable residues (V107, V192 and L111) facilitated the closure of the Gate/L6/CL2 via bulky hydrophobic residue substitutions. PYL3 V108K V107L (corresponding to PYL2 V87L I88K) [17] constitutively inhibited the activity of HAB1 by 20%, while PYL3 V108K V107F (PYL3 V107F corresponding to PYR1 V83F [22]) constitutively inhibited HAB1 activity by 40% (Fig. 3). The V192 and/or L111 mutated into a bulky and hydrophobic residue, such as Phe in PYL3 V108K V107F, which can significantly promote the closure of the Gate/L6/CL2.

PYLs comprise 14 members with highly similar sequences and have evolved and adapted to perceive the ABA signal under different environmental conditions, including drought, cold or heat stresses. Activation of individual or PYR1/PYLs subclass receptors selectively by chemical agonists [37, 38] or genetic methods [39] is critical to revealing the different functions of the large family of receptors. The loss‐of‐function mutation of single PYR1/PYLs may not produce an obvious phenotype because of functional redundancy and lethality at some development stage. The gain‐of‐function mutation can be leveraged to scrutinize individual receptor function *in vivo*. Expression of activated PYL2^CA3^ (corresponding to PYR1^CA3^) in transgenic *Arabidopsis* seeds activates ABA signaling [22]. The common V83F in PYR1^CA3^, PYR1^CA4^ and PYR1^CA4B^ promotes the Gate/L6/CL2 closure, while the remaining two‐ or three‐residue substitution in the C‐terminal helix increases the interactions between PYR1–PP2C interface, which eliminates the original interaction difference between PYR1 and different members of PP2Cs. In this study, the mutation sites of four PYL3 mutants with potent ABA‐independent activation rarely participate in the direct binding of PP2Cs. Thus, they all retain nearly the same crosstalk differences between PYL3 WT and different members of PP2Cs. Therefore, these four PYL3 mutants introduced into transgenic plants will help dissect the functions of PYL3 *in vivo*.

## Conflict of interest

The authors declare no conflict of interest.

## Author contributions

Xingliang Zhang and Xiaoli Zhang conceived and designed the experiments. Xingliang Zhang, YW, CF, XW and WL performed the experiments. Xingliang Zhang, YW and Xiaoli Zhang contributed reagents/materials/analysis tools. Xingliang Zhang and Xiaoli Zhang wrote the paper. All authors analyzed the results and approved the final version of the manuscript.

## Supporting information


**Table S1.** Primers for PCR or site mutation.
**Fig. S1.** The molecular mechanisms of PYL3 recognizing ABA and inhibiting HAB1.
**Fig. S2.** The molecular mechanisms of PYL3 recognizing ABA and inhibiting HAB1.
**Fig. S3.** ITC detected the interactions between PYL3 V192F protein and ABA.
**Fig. S4.** Enzyme digestion identification, prokaryotic expression, purification of ABI1 and ABI2, and inhibition of the four mutated PYL3 on ABI1 and ABI2.
**Fig. S5.** The inhibition of the two mutated PYL9 on HAB1 by the Serine‐Threonine Phosphatase Assay System Kit.

## Data Availability

All data generated or analyzed during this study are included in this published article.
