# Supplementary material for: Potent ABA‐independent activation of engineered PYL3
Source: FEBS Open Bio. 2021 Apr 7;11(5):1428–39. doi: 10.1002/2211-5463.13151 (PMC8091583; doi:10.1002/2211-5463.13151)
Supplement: Supplementary file 1 — Table S1. Primers for PCR or site mutation. Fig. S1. The molecular mechanisms of PYL3 recognizing ABA and inhibiting HAB1. Fig. S2. The molecular mechanisms of PYL3 recognizing ABA and inhibiting HAB1. Fig. S3. ITC detected the interactions between PYL3 V192F protein and ABA. Fig. S4. Enzyme digestion identification, prokaryotic expression, purification of ABI1 and ABI2, and inhibition of the four mutated PYL3 on ABI1 and ABI2. Fig. S5. The inhibition of the two mutated PYL9 on HAB1 by the Serine‐Threonine Phosphatase Assay System Kit. [file FEB4-11-1428-s001.doc]

**Supplementary Information**

**Table S1. Primers for PCR or site-mutation**

| Template | Position or Mutation | F  or  R | Primers' sequence (5'-3') |
| --- | --- | --- | --- |
| PYL3 | 1 | F | **ACT** *ggatcc* ATGAATCTTGCTCCAATCC |
|  | 209 | R | **CACG** *ctcgag* TCAGGTCGGAGAAGCCGT |
| HAB1 | 169 | F | **CT** *ggatcc* GATGAAAATAGTAATCATCTGG |
|  | 511 | R | **CA** *ctcgag* TCAGGTTCTGGTCTTG |
| ABI1 | 104 | F | **CACTCT** *catatg* GATATTACTAGCGAGAAG |
|  | 434 | R | **CA** *ctcgag* TCAGTTCAAGGGTTTG |
| ABI2 | 101 | F | **ACTTG** *catatg* GAGAGTAGAAGTCTGTTTGAGTTC |
|  | 423 | R | **AC** *ctcgag* TCAATTCAAGGATTTGCTCTTGAATTT |
| PYL3 | F81A | F | ATACAAACACGCCATCAAGAGTTGCACCATCAG |
|  |  | R | ACTCTTGATGGCGTGTTTGTATTTGTTTGGATTG |
| PYL3 | F188A | F | TACAAGGATGGCTGTGGATACGGTCGTTAAGTC |
|  |  | R | CGTATCCACAGCCATCCTTGTATCTTCTTCCG |
| PYL3 | V108A | F | GAAGTTAGCGTGGCCTCTGGTCTTCCAGCG |
|  |  | R | AAGACCAGAGGCCACGCTAACTTCCCTTAT |
| PYL3 | V108K | F | GAAGTTAGCGTGAAGTCTGGTCTTCCAGCGTCA |
|  |  | R | TGGAAGACCAGACTTCACGCTAACTTCCCTTAT |
| PYL3 | V108E | F | GAAGTTAGCGTGGAGTCTGGTCTTCCAGCGTCA |
|  |  | R | TGGAAGACCAGACTCCACGCTAACTTCCCTTAT |
| PYL3 | L111F | F | GTGGTCTCTGGTTTTCCAGCGTCAACAAGC |
|  |  | R | GTTGACGCTGGAAAACCAGAGACCACGCTAAC |
| PYL3 | V107F | F | AGGGAAGTTAGCTTCGTCTCTGGTCTTCCAGCG |
|  |  | R | AAGACCAGAGACGAAGCTAACTTCCCTTATAGT |
| PYL3 | V192F | F | TTTGTGGATACGTTCGTTAAGTCGAATCTACAG |
|  |  | R | ATTCGACTTAACGAACGTATCCACAAACATCCT |
| PYL3 V108K | V107L | F | AGGGAAGTTAGCCTGAAGTCTGGTCTTCCAGCGTCAACA |
|  |  | R | TGGAAGACCAGACTTCAGGCTAACTTCCCTTATAGTCCC |
| PYL3 V108K V107L | V192L | F | TTTGTGGATACGCTCGTTAAGTCGAATCTACAG |
|  |  | R | ATTCGACTTAACGAGCGTATCCACAAACATCCTTGT |
| PYL3 V108K V107L | V192F | F | TTTGTGGATACGTTCGTTAAGTCGAATCTACAG |
|  |  | R | ATTCGACTTAACGAACGTATCCACAAACATCCT |
| PYL3 V108K | V107F | F | AGGGAAGTTAGCTTCAAGTCTGGTCTTCCAGCGTCAACA |
|  |  | R | TGGAAGACCAGACTTGAAGCTAACTTCCCTTATAGTCCC |
| PYL3 V108K V107F | V192F | F | TTTGTGGATACGTTCGTTAAGTCGAATCTACAG |
|  |  | R | ATTCGACTTAACGAACGTATCCACAAACATCCT |
| PYL3 V108K V107L | L111F | F | CTGAAGTCTGGTTTTCCAGCGTCAACAAGCGTT |
|  |  | R | TGTTGACGCTGGAAAACCAGACTTCAGGCTAAC |
| PYL3 V108K V107L V192L | L111F | F | CTGAAGTCTGGTTTTCCAGCGTCAACAAGCGTT |
|  |  | R | TGTTGACGCTGGAAAACCAGACTTCAGGCTAAC |
| PYL3 V108K V107L L111F | V192F | F | TTTGTGGATACGTTCGTTAAGTCGAATCTACAG |
|  |  | R | ATTCGACTTAACGAACGTATCCACAAACATCCT |
| PYL3 V108K V107F V192F | L111F | F | TTCAAGTCTGGTTTTCCAGCGTCAACAAGCGTT |
|  |  | R | TGTTGACGCTGGAAAACCAGACTTGAAGCTAAC |

Note: The restriction site bases are shown in small letters and italics. The protective bases for restriction enzyme [recognition](javascript:;) are shown in bold and underlined capital letters. F: Forward, R: reverse.


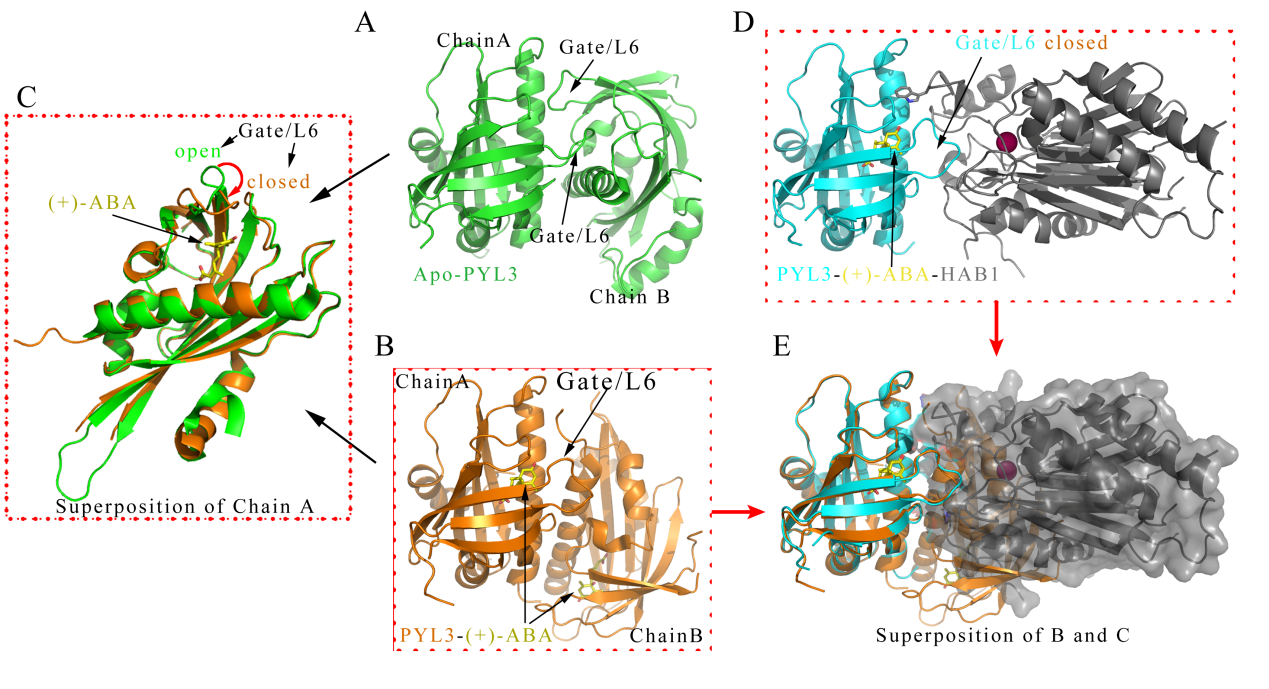


**Figure S1. The molecular mechanisms of PYL3 recognizing ABA and inhibiting HAB1.** **(A)** The crystal structure of apo-PYL3 (PDB: 3KLX). **(B)** The complex crystal structure of ABA-bound PYL3 (PDB: 4DSC). **(C)** Upon binding ABA, the Gate/L6 of PYL3 became closed, displaying the most significant conformational change by superimposing two Chain A in (A) and (B). **(D)** PYL3 recognized ABA and then combined with downstream substrate HAB1 (PDB: 4DSC). **(E)** Superposition of PYL3 in (B) and (D) showed their Gate/L6 conformations were closed.


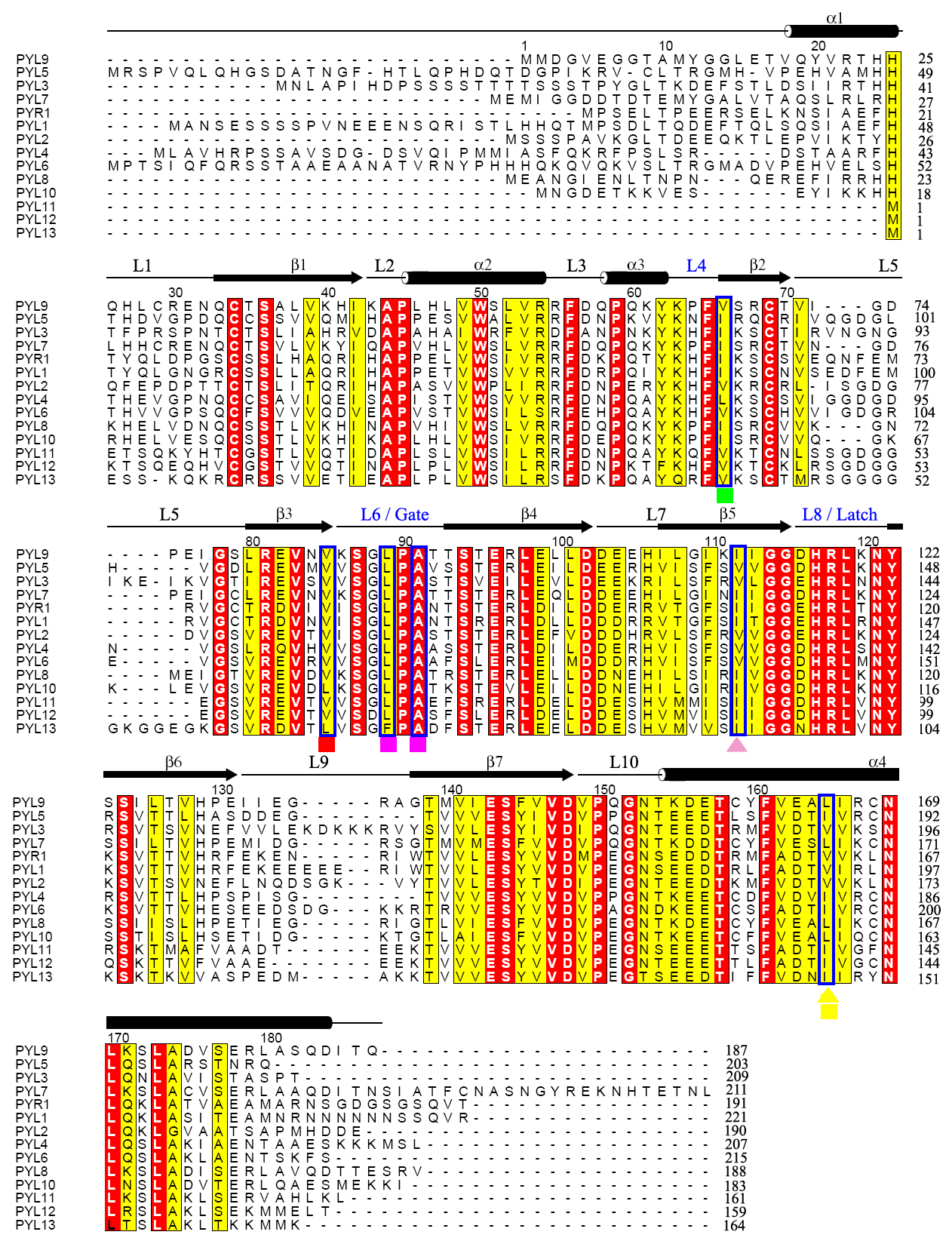


**Figure S2. Primary sequence alignment of PYLs members.** The sequence alignment was generated by ClustalW [[1](#_ENREF_1)]. This figure was made by the program ALSCRIPT [[2](#_ENREF_2)]. Conservation values range from 0-10, where 10 is an identity for the complete alignment. Conservation values above 5 showed increasing similarities in physico-chemical properties. All columns of the alignment for which the conservation value better than 5 were showed in the yellow background for the black character of residues. The identical residues in the red background were represented in white characters. The nomenclature according to the publication, was colored blue. For example, L6 is also known as Gate [[3](#_ENREF_3)] or CL2 [[4](#_ENREF_4)], and L8 is also known as latch [[3](#_ENREF_3)] or CL3 [[4](#_ENREF_4)]. The five residues involved in facilitating the Gate/L6 closure were marked with a rectangle under PYL13.


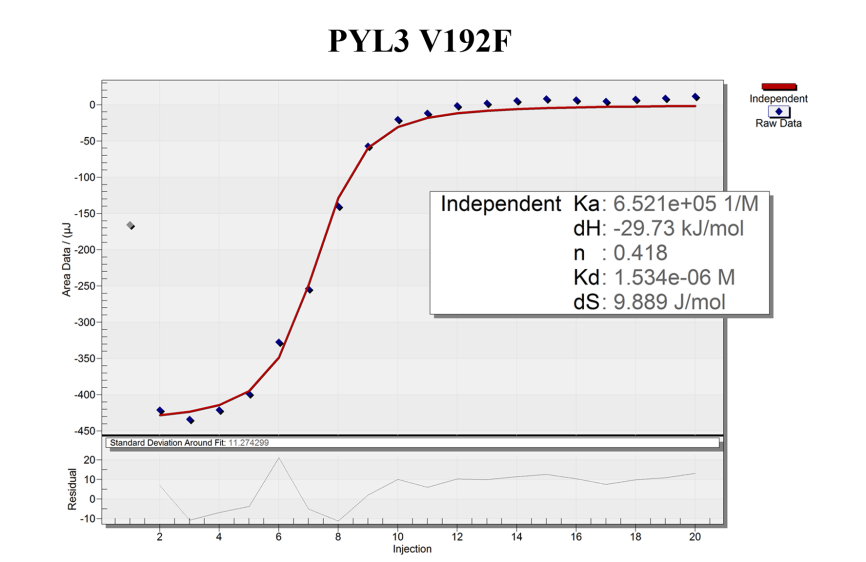


**Figure S3. ITC detected the interactions between PYL3 V192F protein and ABA.**


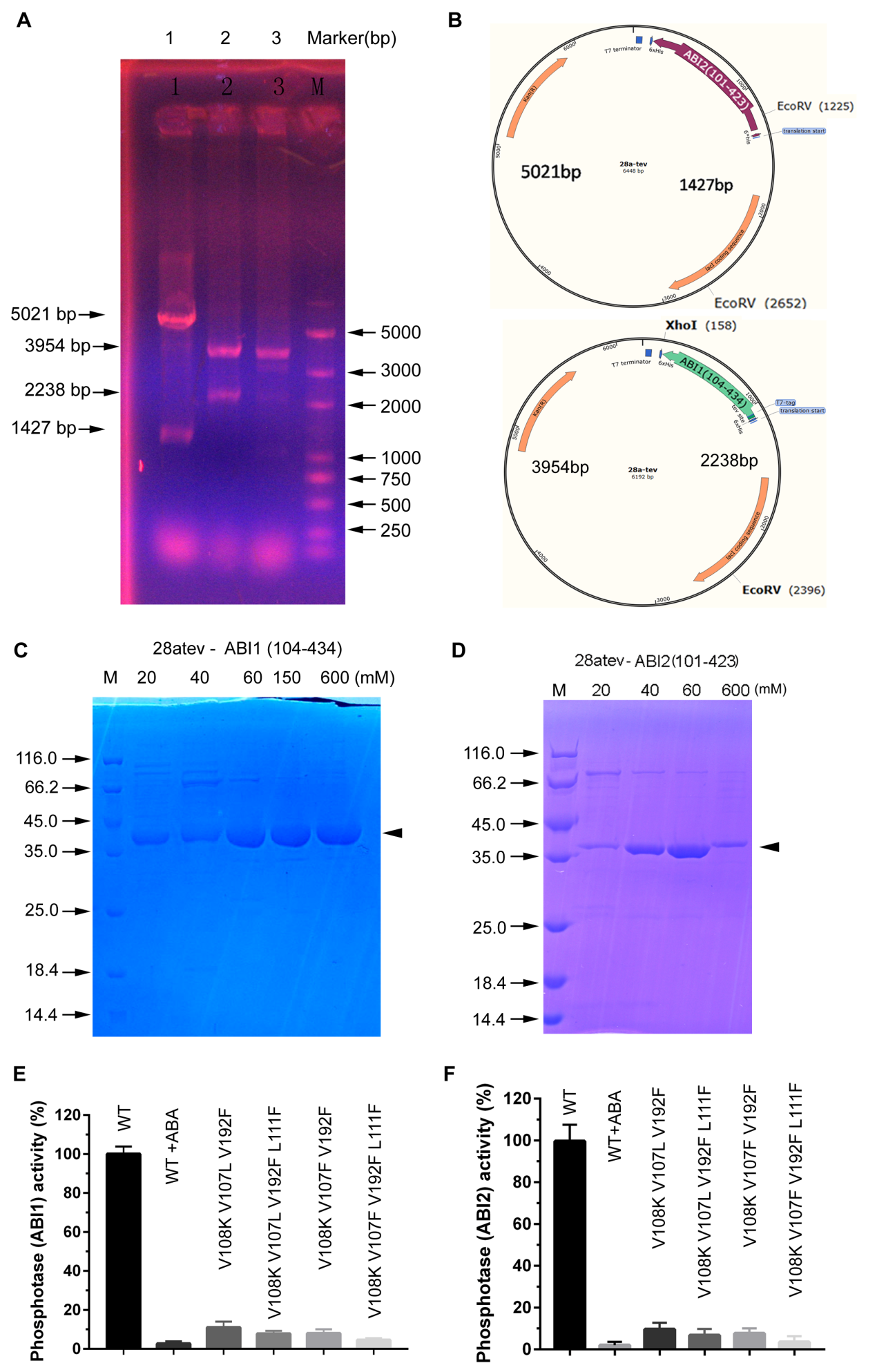


**Figure S4. Enzyme digestion identification, prokaryotic expression, purification of ABI1 and ABI2, and inhibition of the four mutated PYL3 on ABI1 and ABI2. (A)** Enzyme digestion identification of pET-28a-tev-ABI2 (101-423) upon Eco*RV* and Xho*I* (Lane 1) as well as pET-28a-tev-ABI1 (104-434) upon Eco*RV* (Lane 2 and 3). **(B)** The theoretical sizes of fragments for pET-28a-tev-ABI1(104-434) and pET-28a-tev-ABI2 (101-423) after restriction enzyme digestion. Two recombinant plasmids in Lane 1 and Lane 2 were chosen for DNA sequencing identification. **(C-D)** 6×His-tagged ABI1 (104-434) protein was gradiently eluted from Ni column by the 20, 40, 60, 150, and 600 mmol·L^-1^ imidazole. 6×His-tagged ABI2 (101-423) protein was gradiently eluted from Ni column by the 20, 40, 60, and 600 mmol·L^-1^ imidazole. **(E-F)** The inhibition of the four mutated PYL3 on ABI1 and ABI2 by the Serine-Threonine Phosphatase Assay System Kit. Data were expressed as Mean±SEM.


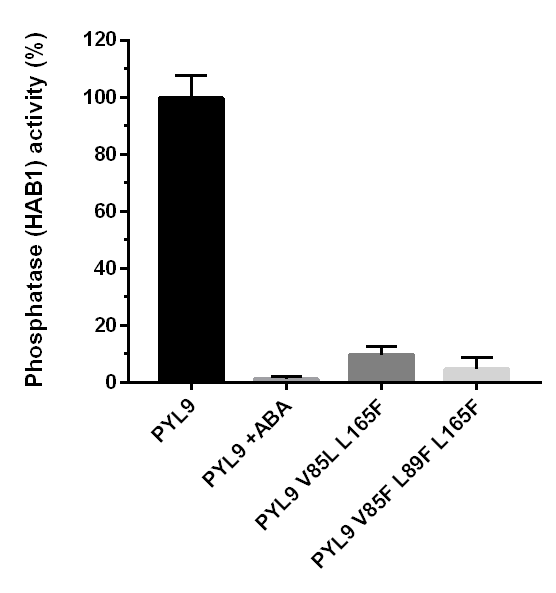


**Figure S5.** The inhibition of the two mutated PYL9 on HAB1 by the Serine-Threonine Phosphatase Assay System Kit. Data were expressed as Mean±SEM.

# Supplementary References

1. Larkin MA, Blackshields G, Brown NP, Chenna R, McGettigan PA, McWilliam H, et al. Clustal W and Clustal X version 2.0. Bioinformatics. 2007;23(21):2947-8. doi: 10.1093/bioinformatics/btm404.

2. Barton GJ. ALSCRIPT: a tool to format multiple sequence alignments. Protein Eng. 1993;6(1):37-40. doi: 10.1093/protein/6.1.37.

3. Melcher K, Ng LM, Zhou XE, Soon FF, Xu Y, Suino-Powell KM, et al. A gate-latch-lock mechanism for hormone signalling by abscisic acid receptors. Nature. 2009;462(7273):602-8. Epub 2009/11/10. doi: nature08613 [pii]

10.1038/nature08613. PubMed PMID: 19898420; PubMed Central PMCID: PMC2810868.

4. Yin P, Fan H, Hao Q, Yuan X, Wu D, Pang Y, et al. Structural insights into the mechanism of abscisic acid signaling by PYL proteins. Nat Struct Mol Biol. 2009;16(12):1230-6. Epub 2009/11/07. doi: nsmb.1730 [pii]

10.1038/nsmb.1730. PubMed PMID: 19893533.
